# New Appliances and Things Medical

**Published:** 1902-02-01

**Authors:** 


### NEW APPLIANCES AND THINGS MEDICAL.

[We shall be glad to receive, at our Office, 28 & 29 Southampton Street, Strand, London, W.C., from the manufacturers, specimens of all new preparations and appliances which may be brought out from time to time.]

### HORLICK'S MALTED MILK AND LUNCH TABLOIDS.

(Horlick & Co,, 34 Farringdon Road, London, E.C.)

Horlick's Malted Milk was among the first of the desiccated milk preparations to recommend itself by its genuine merits to the notice of doctors and nurses. Analysis shows that this malted milk contains a large proportion of soluble carbo-hydrates and the dried constituents of cows' milk. The soluble carbo-hydrates are prepared from wheat which has been acted upon by barley malt and its contained diastatic ferment, and therefore consists of sugar alone, no starch remaining to upset the infantile digestion. Practical experience has proved over and over again that infants whose digestion has been upset by injudicious feeding recover completely on an exclusive diet of Horlick's malted milk. Although predigested foods of the character of Horlick's Malted Milk are invaluable for temporary use or for an emergency, it must be remembered that they cannot be employed indefinitely without the addition of some fresh food, such as fresh cows' milk, meat juice, or the juice of fruit, otherwise scorbutic symptoms will ultimately supervene. For use to tide over a difficulty, or in combination with some fresh form of food, this malted milk will prove invaluable. Horlick's lunch tabloids, which are composed of the malted milk compressed in the form of small round biscuits, are useful as a concentrated form of nutritious and energy-supplying food. Bicyclists or soldiers on the march will thoroughly appreciate the sustaining qualities of these tabloids: in a minimum of bulk is contained a maximum of nutritive elements, the very best which can be derived from milk and wheat.

